# Three-dimensional evaluation of the cortical and cancellous bone density and thickness for miniscrew insertion: a CBCT study of interradicular area of adults with different facial growth pattern

**DOI:** 10.1186/s12903-023-03440-x

**Published:** 2023-10-13

**Authors:** Mahvash Hasani, Saeed Afzoon, Maryam Karandish, Mina Parastar

**Affiliations:** 1 Department of Oral and Maxillofacial Radiology, School of Dentistry, Shiraz University of Medical Sciences, Shiraz, Iran; 2 School of Dentistry, Shiraz University of Medical Sciences, Shiraz, Iran; 3 Department of Orthodontics, School of Dentistry, Shiraz University of Medical Sciences, Shiraz, Iran

**Keywords:** CBCT, Bone density, Bone thickness, Growth pattern, Orthodontic mini-screws

## Abstract

**Aim:**

The purpose of this study was to evaluate the effect of the density and the thickness of the cortical and the cancellous bone at selected inter-radicular areas in subjects with different facial growth patterns using cone beam computed tomography (CBCT) in order to choose the optimal area for miniscrew insertion.

**Materials and methods:**

From 150 CBCT scans, 45 scans were included in the study. The subjects were categorized into three groups based on their skeletal growth pattern according to SN-GoMe angle and facial height index. Cortical and cancellous bone density and thickness were measured at the selected inter-radicular areas.

**Results:**

Compared to the other two groups, the hyperdivergent group had thinner cortical bone in the anterior region of the maxilla between the central and the lateral incisors on the buccal side at 4 mm from the alveolar crest (P-value: 0.012) and on the palatal side at 7 mm from the alveolar crest (P-value: 0.030). Cancellous bone density values in these areas were higher in subjects with hypodivergent and hyperdivergent growth pattern. Furthermore, in hyperdivergent group less dense cortical bone in the posterior region of the maxilla on the palatal side between the second premolar and the first molar (p-value: 0.020) and on the buccal side between the first molar and the second molar (p-value: 0.038 & 0.047) was observed. No significant differences were found in the mandible between the three groups. No significant differences were found between the male and the female subjects.

**Conclusion:**

Hyperdivegents presented thinner cortical bone in the anterior of the maxilla between the central and the lateral incisors. Less dense cortical bone was found between maxillary second premolar and first molar on the palatal side and also between the maxillary first molar and the second molar on the buccal side in this group too. Normal showed higher density values in the posterior of the maxilla compared to the other two groups. No significant differences were found among three groups in mandible.

## Introduction

Appling force to the teeth during orthodontic treatment generates an equal force with the same magnitude in the opposite direction which can result in unwanted tooth movement [1]. Undesirable tooth movement and the opposite forces must be controlled to reach the optimum treatment result [1, 2]. Anchorage in orthodontics is defined as controlling the unwanted tooth movement [3].Different appliances are designed to play the role of anchorage in order to facilitate tooth movement [4].They include palatal implants, miniplates and mini-screws and etc. [5, 6]. Most orthodontists prefer to utilize minscrews as they are convenient to use, easily accepted by patients and cost-effective [3, 7, 8].

Miniscrews may loosen during orthodontic treatment; therefore, the stability of the miniscrews is essential to enhance the success rate of skeletal anchorage [9, 10]. The stability of miniscrew relies on multiple factors such as: the thickness and density of the cortical bone, the depth of the inter-radicular space, the soft tissue features, the physical characteristics of mini screw and the method of miniscrew insertion [11–14].

Among all these factors, the cortical bone thickness plays the most important role in miniscrew initial stability and it increases long-term success of orthodontic treatment [15]. It is worth mentioning that the cancellous bone volume is also influential when cortical bone is not thick enough [7, 15].

Bone density is another important factor that affects the amount of the miniscrew in contact with the bone, resistance capability and rate of tooth movement [16–18]. During the early stage of miniscrew insertion, bone density is a crucial factor for stationary anchorage of mini-screws specially in the areas with insufficient cortical bone thickness [16, 19]. The primary retention of the miniscrews is achieved by mechanical contact between the bone and miniscrew rather than osteointegration [19, 20]. Both cortical and cancellous bone density are reported to be related to the stability of mini-screws [16, 21].Studies conducted with micro-CTs indicate that cancellous bone is an essential factor that influences the primary stability of mini-screws [21].

The structure of facial bones and muscles is dominantly controlled by genetic factors [22]. Additionally, functional loads significantly affect the craniofacial morphology [10]. craniofacial skeleton and muscles can affect the growth pattern, oral function and the vertical facial dimension [23]. Facial growth pattern has been reported to be related to masticatory muscles development [24]. Moreover, it has been revealed that different facial types influence the cortical bone shape, thickness and mineralization [25].

Bone structure is considered to have a close connection with the facial growth pattern [26]. Studies revealed that patients with hyperdivergent growth pattern are at risk of miniscrew failure since they possess thinner and less dense dento-alveolar bone [27]. Another study indicates that lower incisor cancellous bone support is significantly associated with hyperdivergent growth pattern [28]. It has been suggested that the hypodivergent and normal facial growth patterns are associated with thicker lower incisor bony support compared to hyperdivergent patients [28]. As hyperdivergent patients have thinner cortical bones, percussions should be taken when inserting minscrews [29].

The combined effect of the density and the thickness of the cortical and the cancellous bone in the optimal sites in the maxilla and the mandible for the placement of mini screws in subjects with different facial types is a topic little discussed [30]. This CBCT based study was designed with the hypothesis that whether there is any differences in the quality and quantity of the optimal sites for inserting miniscrews in the anterior and posterior regions of the maxilla and the mandible in respect of different growth patterns.

## Materials and methods

This study is approved by the research ethics committee of the university (Grant#IR.SUMS.REC.1397.984). 150 full face CBCT scans of patients referred to oral and maxillofacial radiology department of Shiraz Dental School were retrieved using non-probability convenience sampling. The informed consent was obtained from all subjects. Patients with systemic disease (such as: Hypothyroidism, Rheumatoid Arthritis, Diabetes, etc.), previous or current orthodontic treatment, obvious periodontal disease, evidence of previous trauma, missing and severely ectopic teeth were excluded from the study.

**Fig. 1:**
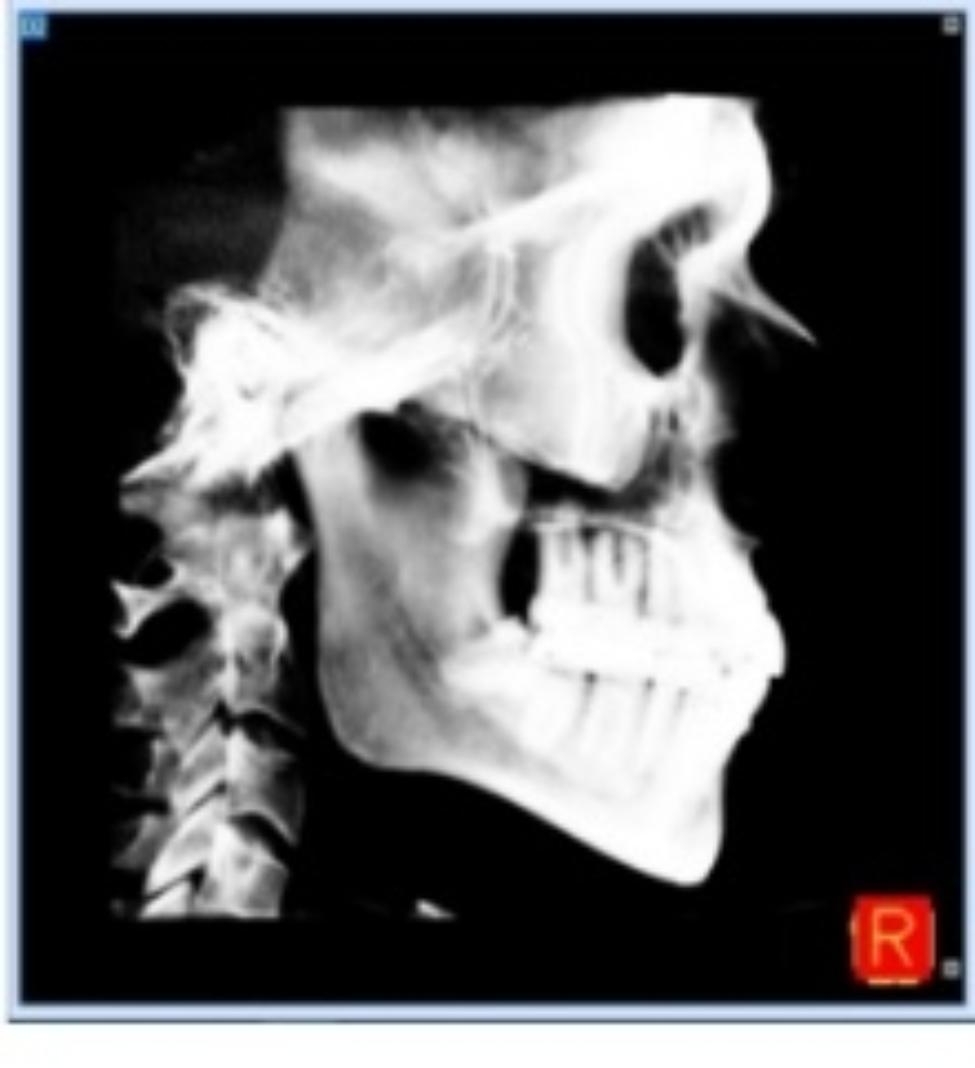
A CBCT synthesized lateral cephalograms

The CBCT images had been obtained using the FPD-based CBCT (New TomVGi, QRSrL, Italy). The CBCT scan had been set with an exposure time of 3.6 s, 110kVp and 15 cm* 15 cm field of view. The images were analyzed using NNT viewer software. In order to analyze the subjects’ facial growth pattern, CBCT-synthesized lateral cephalograms were generated (Fig. 1). By analyzing the images 45 scans were ultimately selected for further investigations.

CBCT-synthesized lateral cephalograms were saved as JPEG images and imported to Onyx Ceph™ software (version 2.7.7, image instrument, Chemints, Germany) for the purpose of assessing the following cephalometric measurements: facial height index (posterior facial height divided by anterior facial height multiplied by 100), SN-GoGn (angel between sella-nasion and gonion-menton). According the cephalometric analysis subjects were divided into three facial type categories: hyperdivergent group formed from 11 women and 2 men (SN-GoGn > 39° and facial height ≤ 59%), normal (group consisting of 13 women and 4 men (28° ≤ SN-GoGn ≤ 39° and 59% ≤ facial height index ≤ 63%) and hypodivergent group including 10 women and 5 men (SN-GoGn < 28°and facial height index ≥ 63%).

The cancellous and the cortical bone thickness and density of the inter-radicular area between the maxillary central and the lateral incisors, between the maxillary second premolar and first molar, between the maxillary first molar and second molar and in the mandible between the mandibular lateral incisor and canine, between the mandibular second premolar and first molar and between the mandibular first molar and second molar were selected for measurement. The mentioned sites are of the most frequently used sites for mini screw insertion.

**Fig. 2:**
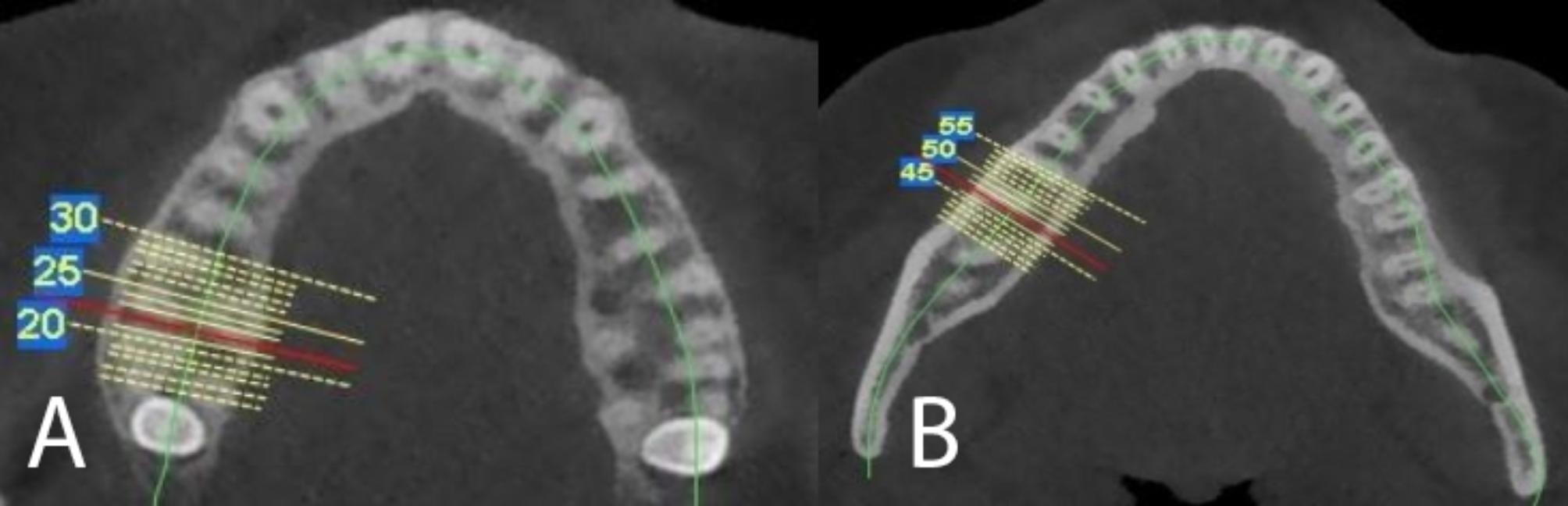
Axial section of the maxilla (**A**) and axial section of the mandible (**B**)

**Fig. 3:**
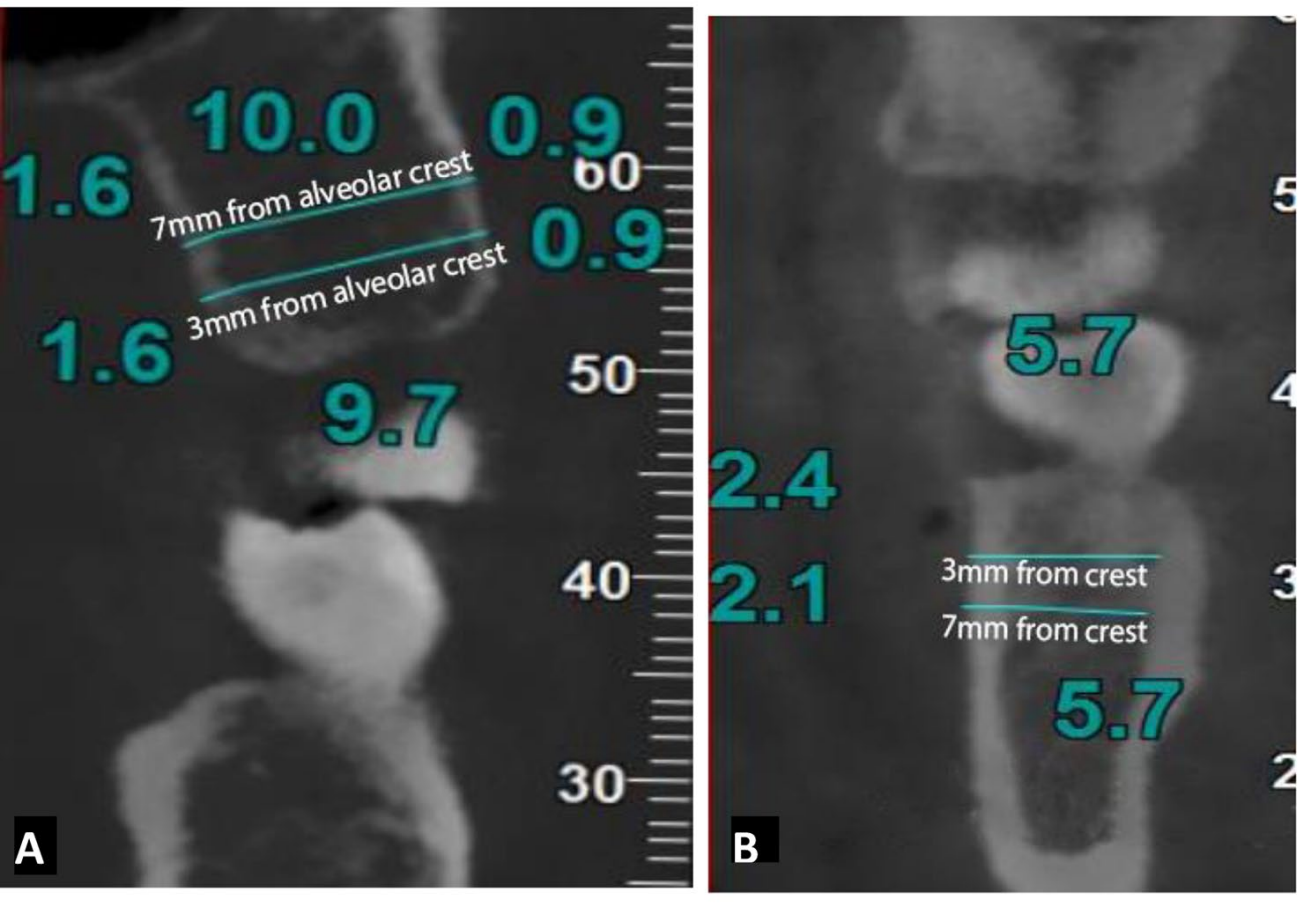
**A)** Corrected coronal section through the inter-radicular area between upper left first and second molars With the measurements of inter-radicular buccal cortical plate thickness at 4 mm (IBCBT4) and buccal cortical plate thickness at 7 mm (BCBT7) apical to the crest of the alveolar bone. inter-radicular palatal cortical plate thickness at 4 mm (IPCBT4) and inter-radicular palatal cortical plate thickness at 7 mm (IPCBT7) apical to the crest of the alveolar bone. inter-radicular cancellous bone thickness at 4 mm (ICBT4) and inter-radicular cancellous bone thickness at 7mm (ICBT7) apical to the crest of the alveolar bone. **B**) Coronal section through the inter radicular area between lower right second premolar and first molar with the measurements.

The measurement sites were determined on the multi-planar images. We marked the middle of the inter-radicular area on the axial sections(Fig. 2). In the corrected coronal view, the thicknesses and the densities of the cortical (labial/buccal and palatal) and cancellous bone at 4 and 7 mm from the alveolar bone crest were measured (Fig. 3). The Cortical and cancellous bone densities in the interdental areas were measured using Hounsfield units (HU). The cancellous bone density of the maxilla was measured at the one third of its total thickness near the buccal and palatal cortical bone where is almost the end of the length of the mini screw and the mean values were recorded. In the mandible the density value near the buccal cortex was only considered because of lingual cortex limited use for mini-screw placement.

### Statistical analysis

Data were presented by mean and standard deviation. One-way ANOVA analysis was used to compare the mean values of densities and thicknesses of the three groups. Subsequently, pair-wise comparisons were carried out using Tukey and LSD post hoc tests to discover the differences. The data analysis was performed with IBM SPSS statistics version 25 for windows and P < 0.05 was considered significant in this study.

## Results

In the anterior region between the maxillary central and lateral incisors, the mean value of the buccal inter-radicular cortical bone thickness at 4 mm from the alveolar crest (IBCBT4) was lower for hyperdivergent group than the other two groups. The hypodivergent and hyperdivergent group showed higher values for inter-radicular cancellous bone density at 4 mm from alveolar crest (ICBD4) in the mentioned site in compare to normal group. the mean value of the inter-radicular palatal cortical bone thickness at 7 mm from the alveolar (IPCBT 7) at the selected sites was lower for hyperdivergent group than normal and hypodivergent group (Table 1).

In the posterior region, between the maxillary second premolar and first molar, the mean values of the cortical and the cancellous bone thickness and density did not show any statistically significant differences. However, buccal inter-radicular cortical bone thickness at 4 mm from the alveolar crest (IBCBD4) of the mentioned site showed higher values in normal group than the other two groups. The mean value of the inter-radicular palatal density at 7 mm from the alveolar crest (IPCBD7) was lower for hyperdivegent group than normal group in this site (Table 1).

Between the maxillary first molar and second molar, the mean value of the buccal inter-radicular cortical bone thickness at 4 mm from the alveolar crest (IBCBD4) was greater for normal group in compare to the other two groups. The mean value of inter-radicular buccal cortical bone density at 7 mm from the alveolar crest (IBCBD7) of hyperdivergent group was lower than normal group in this site (Table 2).

Regarding the cancellous bone thickness in the anterior region and cortical and cancellous bone thickness in the posterior region of the maxilla, no significant differences were found between the three groups. Considering the inter-radicular cortical and cancellous bone thickness and density between the lateral incisor and canine, between the second premolar and first molar and between the first molar and the second molar at 4 and 7 mm from the alveolar crest in the lower arch, no statistically significant differences were found among three facial types (Tables 2 and 3). The results of this study showed no statistically significant differences between male and female subjects.

**Table 1:** Bone characteristics measurements between upper central and lateral incisors in relation to facial types

Bone sites/Growth pattern	IBCBT4	IBCBT7	IBCBD4	IBCBD7	ICBBT4	ICBT7	ICBD4	ICBD7	IPCBT4	IPCBT7	IPCBD4	IPCBD7
hyperdivergent	1.180 ± 0.230	1.376 ± 0.279	952.807 ± 221.635^b^	1053.769 ± 150.503	7.623 ± 1.238	7.203 ± 1.443	402.615 ± 146.438	340.807 ± 171.259	1.461 ± 0.339	1.530 ± 0.338	661.000 ± 169.408	640.423 ± 115.307^b^
hypodivergent	1.213 ± 0.240	1.493 ± 0.999	942.933 ± 148.009^b^	1080.533 ± 181.459	8.346 ± 1.383	7.876 ± 1.975	343.100 ± 139.335	306.966 ± 106.411	1.386 ± 0.251	1.816 ± 0.695	704.633 ± 136.180	712.666 ± 135.923^a’b^
normal	1.320 ± 0.215	1.417 ± 0.236	1121.735 ± 122.326^a^	1197.852 ± 193.417	7.970 ± 1.286	7.808 ± 1.282	471.088 ± 203.389	436.588 ± 174.566	1.388 ± 0.240	1.588 ± 0.310	727.558 ± 173.956	778.705 ± 130.467^a^
p-value	0.012*	0.341	0.778	0.167	0.957	0.582	0.010*	0.154	0.165	0.030*	0.299	0.506

*^a’b^ Same superscript letters indicate no significant difference and different letters are statistically significantly different

*: indicates significant difference between groups

**Table 2:** Bone characteristics measurements between second premolar and first molar

Bone sites/Growth pattern	IBCBT4	IBCBT7	IBCBD4	IBCBD7	ICBBT4	ICBT7	ICBD4	ICBD7	IPCBT4	IPCBT7	IPCBD4	IPCBD7
hyperdivergent	1.180 ± 0.230	1.376 ± 0.279	952.807 ± 221.635^b^	1053.769 ± 150.503	7.623 ± 1.238	7.203 ± 1.443	402.615 ± 146.438	340.807 ± 171.259	1.461 ± 0.339	1.530 ± 0.338	661.000 ± 169.408	640.423 ± 115.307^b^
hypodivergent	1.213 ± 0.240	1.493 ± 0.999	942.933 ± 148.009^b^	1080.533 ± 181.459	8.346 ± 1.383	7.876 ± 1.975	343.100 ± 139.335	306.966 ± 106.411	1.386 ± 0.251	1.816 ± 0.695	704.633 ± 136.180	712.666 ± 135.923^a’b^
normal	1.320 ± 0.215	1.417 ± 0.236	1121.735 ± 122.326^a^	1197.852 ± 193.417	7.970 ± 1.286	7.808 ± 1.282	471.088 ± 203.389	436.588 ± 174.566	1.388 ± 0.240	1.588 ± 0.310	727.558 ± 173.956	778.705 ± 130.467^a^
p-value	0.216	0.877	0.005*	0.067	0.351	0.479	0.111	0.058	0.719	0.248	0.534	0.020*

*^a’b^ Same superscript letters indicate no significant difference and different letters are statistically significantly different

*: indicates significant difference between groups

**Table 3:** Bone characteristics measurements between upper first molar and second molar

Bone sites/Growth pattern	IBCBT4	IBCBT7	IBCBD4	IBCBD7	ICBBT4	ICBT7	ICBD4	ICBD7	IPCBT4	IPCBT7	IPCBD4	IPCBD7
hyperdivergent	1.253 ± 0.183	1.365 ± 0.191	927.692 ± 183.248^b^	1027.961 ± 145.257^b^	10.176 ± 1.186	10.084 ± 1.054	517.153 ± 218.670	426.769 ± 240.937	1.334 ± 0.278	1.496 ± 0.289	756.807 ± 140.699	764.269 ± 129.838
hypodivergent	1.260 ± 0.254	1.586 ± 0.348	935.466 ± 160.522^b^	1051.133 ± 160.290^a’b^	11.290 ± 1.419	11.196 ± 1.496	504.266 ± 212.653	394.733 ± 211.267	1.476 ± 359	1.573 ± 0.472	753.366 ± 146.448	741.400 ± 139.333
normal	1.264 ± 0.235	1.491 ± 0.347	1062.558 ± 140.412^a^	1154.088 ± 133.835^a^	10.700 ± 1.349	10.547 ± 1.554	554.441 ± 269.704	468.617 ± 261.520	1.308 ± 0.280	1.529 ± 0.273	758.852 ± 162.487	759.176 ± 166.946
p-value	0.992	0.184	0.038*	0.047*	0.098	0.122	0.825	0.685	0.280	0.847	0.995	0.909

## Discussion

Among different factors that play a role in miniscrews success rate, cortical bone thickness and density are of major importance. The miniscrews retention relies on mechanical retention rather than osteointegration [7, 20]. Furthermore, it is reported that the cancellous bone density is of great significance for miniscrew insertion in the absence or presence of cortical bone and in this regard its thickness becomes important when there is a thin cortical bone [7, 31]. Therefore, knowledge of the bone characteristics enables the clinician to develop appropriate anchorage strategies.

It has been reported that there is a relationship between different vertical facial dimensions and bone morphology [24, 32]. In this regard, this study aimed to compare the buccal and the palatal cortical and cancellous bone thickness and density of three different facial patterns using CBCT images of the patients at the selected sites for mini-screw placement.

According to the results of this study, the hypothesis was accepted to some extent. In our study, hyperdivergent subjects had thinner buccal cortical bone thickness at 4 mm from the alveolar crest and palatal cortical bone thickness at 7 mm from the alveolar crest between maxillary central and lateral incisors in compare to hypodivergent and normal subjects. Regarding the bone thickness and density no significant differences were found in the mandible among three groups.

Different studies measured the alveolar bone thickness and height in the tooth bearing area of the arch in both jaws. Some studies reveal that hyperdivergent group possess significantly thinner alveolus in the anterior region while no differences were found in the posterior region of the maxilla which is similar to our findings. However, in contrast to our study, hyperdivergent group demonstrated thinner alveolus at almost all sites in the lower arch [24, 33]. Some studies only evaluate the influence of the bone thickness for miniscrew insertion regardless of its density, however, we evaluated the combined effect of density and thickness in the interradicular area commonly selected for miniscrew insertion [24]. Another study revealed that alveolar ridge thickness measurements were greater at all sites for hypodivergent subjects than the hyperdivergent group [34].

Similar to our results, Horner et al. used the sagittal view of interradicular sites 5 mm from the alveolar crest to show that in hypodivergent group on buccal and palatal sides in both jaws the cortical bone is thicker [24]. The only location in the maxilla which is not significantly thicker in the hypodivergent subjects was the buccal site between the first molar and the second premolar. In the mandible, only the buccal sites between the molars and the premolars, and the lingual sites between the second premolar and the first molar showed statistically significant group differences [34].

Some studies examined the correlation between the arch form and facial form [23, 26, 35]. Chaturvedi et al. investigated the relationship between the face form, the arch form and the cortical bone thickness and they found that both the face form and the arch form had significant effect on the cortical bone thickness [23].

Han et al. assessed the relationship between three growth patterns and mandibular posterior tooth and alveolus bone morphology. They reported that patients with horizontal growth pattern possess thicker cortical bone, however, no relationship was found between growth pattern and mandibular cancellous bone thickness in this area [32].

Hoang et al. found thicker alveolus in the anterior region of the mandible at the apex of the root and at the level of the alveolar crest in hyperdivergent patients [36].Qu et al. measured lower incisor cancellous bone thickness (LICBT) at the level of the root apex and found thinner LICBT in hyperdivergent patients [36].This is in contrast to the results of the current study. Our different results might be due to the different measurement sites.

In our study, higher cortical bone density in the posterior region of the maxilla in normal group was observed. It was revealed with our study that in the hyperdivergent group there is a less dense bone on the palatal side between the second premolar and the first molar and on the buccal side between the first molar and the second molar.

Ozdemir et al. [37] study evaluated cortical bone density from the distal aspect of the canine to the mesial aspect of the second molar at 4 mm from the alveolar crest in the three facial growth pattern categories and found that hyperdivergent patients have unfavorable cortical density on the maxillary buccal side. We encountered the same results only between upper first and second molars. They suggested that for the hyperdivergent patients, maxillary palatal side is a favorable site for miniscrew insertion. With reference to their study, it was revealed that in three facial growth pattern groups, the maxillary buccal side can be chosen as an optimal site for mini-implant insertion. Considering the result of our study, we suggest the palatal side is a suitable site for mini-screws insertion in hyperdivergent patients.

According to Ozdemir et al. [37] Subjects with hyperdivergent facial type demonstrated lower values for bone density on the buccal side of the mandible, however, we did not find any differences among the three groups.

Li et al. [30] measured inter-radicular buccal cortical bone thickness (IBCBT), inter-radicular cancellous bone density (ICBD) and inter-radicular cancellous bone density (ICBD) at 3 mm apical to the alveolar bone crest of both jaws. They measured the density of the cancellous bone at buccal and lingual sites located 2.0 mm inside the cortical bone plates and at the central site. ICBD is determined by the mean of the three measured values. But we used a different method for measuring the ICBD. Similar to our results, considering IBCBT measurements of the maxilla at 5–6 (5–6 representing the second premolar and first molar) they reported no significant differences among three facial growth patterns groups. However, BCBT of the mandibular measurements at 5–6, 6–7 and the maxillary cancellous bone density (CBD) measurements at 5–6,6–7 were significantly higher in hypodivergent group than the other two groups. The hypodivergent BCBD measurements at 1–1 was significantly higher than the normal. The hypodivergent and hyperdivergent BCD measurements at 1–2 were significantly higher than the normal group. The CBD measurements for normal group at 1–2 were significantly higher than the hyperdivergent group.

In our study, no significant difference was found between genders which agrees with the previous studies [19, 38, 39]. We measured bone characteristics at selected sites for mini-implant placement which were proved to be the optimal sites by previous studies [40].

The use of CBCT for evaluation of bone density as a guide for miniscrew insertion is a strength point in the study; On the other hand, the questionable diagnostic capacity of CBCTs in converting GVs to HU as was depicted by Eguren et al. [41]due to the lack of clinical studies was of our limitation.

## Conclusion

Hyperdivegent group presented thinner cortical bone in the anterior of the maxilla between the central and the lateral incisors compared to normal and hypodivergent group. Less dense cortical bone was found between maxillary second premolar and first molar on the palatal side and also between the maxillary first molar and the second molar on the buccal side in hyperdivergent group. Normal group showed higher density values in the posterior of the maxilla compared to the other two groups. No significant differences were found among three groups in the mandible. Therefore, clinicians should be aware of the probability of the thin cortical bone in the anterior of the maxilla and less dense cortical bone plates in the posterior of the maxilla which both can increase the risk of mini-implants failure in hyperdivergent subjects. On the basis of present study, we recommend taking precautions in hyperdivergent patients while inserting mini-screws in the anterior of the maxilla because of the existence of the thinner cortical bone. Also, in the posterior of the maxilla as it seems the bone to be less dense in this area mini screw should be inserted with care. It is suggested to conduct further studies with larger sample size to clarify such an issue.

## Data Availability

The datasets used and/or analyzed during the current study are available from the corresponding author on reasonable request.
